# Medical and Surgical Society of Baltimore

**Published:** 1880-03

**Authors:** 


### MEDICAL AND SURGICAL SOCIETY OF BALTIMORE.

---

*Management of Lying-in Women.* Dr. Murray: In opening the discussion on the management of lying-in women, or, more properly, the attention required of the physician in a case of natural labor, for the time is too short to consider any of the complicated cases sometimes met with, I shall follow the beaten track, and note my personal experience as we consider the different points. First, I make an examination to discover whether labor has commenced or not; to ascertain the presentation, the condition of the soft part and the diameters of the pelvis. If the result of the examination is favorable, I direct attention to the minutiæ of the room. If the circumstances of the people will allow, I use a separate bed upon which to deliver, and have her removed to the regular lying-in bed as soon as the process is complete and the soiled clothes removed. I see that a good sharp pair of scissors and a suitable cord are ready. I make no further examinations until the head comes down to the perineum. Frequent examinations are unnecessary and harmful. Sometimes delivery is retarded by the anterior lip of the uterus becoming puffy, and not slipping up over the head of the child, and by keeping it up we may aid matters considerably. I usually preserve the bag of waters, except in those cases in which the womb seems to be unable to contract properly on its contents on account of the excessive amount of liquor annii; then I rupture it, and labor proceeds naturally.

*The Placenta.*—In the Rotunda Hospital, Dublin, where I studied midwifery, it was the rule to make no attempt to deliver the placenta for one hour after the birth of the child, but, on the whole, I do not think this good practice.

My rule is, if, after expulsion of the child, the womb remains large, no hemorrhage, to stimulate contractions by frictions over the abdomen, wetting the hand with cold water and placing it on the abdomen, etc.; but I do not make any attempt at delivery. If the womb is contracted, I remove the placenta in the usual manner. Generally I find the afterbirth partially in the vagina, whence it can be removed by gentle traction. If it be adherent, I introduce my hand into the uterus and detach it. The introduction of the hand is not painful, as the parts are numbed by the passage of the child.

*Ergot.*—I am afraid to give ergot before the delivery of the placenta. Never give it for inertia, although all else seem favorable. I do not use it before the delivery of the child, lest something may occur to prevent delivery, and if so, the tetanic contractions may sacrifice the child's life. Before the plancenta is delivered I am afraid to give it, lest it produce hour-glass contractions. After delivery of the placenta I always administer a full dose, to produce firm contractions, to prevent hemorrhage and to prevent after pains.

*Chloroform.*—It is pretty well settled that it is good practice to give it, and so physicians do so invariably. I do not give it as a rule. I am not afraid of it. In tedious first labors I use it, but always follow it by ergot. I always have all soiled clothes and pads removed immediately after delivery. The movements required are less injurious than allowing these things to remain.

*Binder.*—I think it is useful, and never omit to put it on; I never feel that I have quite done my duty if I do not. It should be broad, extending from the trochanter major to the ensiform cartilage, and securely pinned by not less than four pins. If put on properly it will not slip, and can be worn a week without being displaced. The bandage frequently used, which is about six inches wide, is worse than useless. The advantages are that it gives support to the abdominal viscera, promotes uterine contraction in an indirect way, by counteracting the tendency to expansion, makes the woman more comfortable, and prevents pendulous bellies, which I think, are becoming more common on account of the disuse of the bandage.

*Vaginal Washes.*—These are very desirable, from a hygienic standpoint, and particularly when the lochia is decomposed and stinks. If a disinfectant is needed, I think the best is permanganate of potash. If the lochia is suppressed, hot vaginal injections do more to promote its return than any other remedy.

*Nipples.*—The child should be put to the breast as soon as possible. By so doing the congestion and engorgement of the gland is relieved, and uterine contraction promoted. A cracked and fissured nipple gives a great deal of trouble. Previous attention would prevent many cases, but frequently no opportunity is afforded the physician to prepare them for their duty. After the child has nursed, I direct the nurse to squeeze out the milk from the nipple, dry it carefully with a soft cloth, and dust it with some inert powder, as lycopodium.

*Getting Up.*—I never allow them to get up under nine days, and if I could keep them in bed twenty-five days I would be better pleased. The recommendation of Dr. Goodell on this point does not influence me. I cannot see how it can be otherwise than disastrous. I think it is a great mistake, and produces work for our gyæncological friends. The vaginal douche will remove the discharges better than getting up will.

*Bowels.*—If the rectum be loaded I order an injection immediately after making the first vaginal examination. The conventional third day purge is unnecessary, and facilitates the absorption of the septic matter.

*Diet.*—The diet should be liberal—rich soups, beef, etc., unless some contraindication. I think milk fever is a chimera, and due to other causes. Ventilation is very important.

*Infant.*—I never allow any attention to the child until after the mother has been properly cared for. I do not allow it to be washed, as, on account of the great change in temperature, it becomes chilled, and corysa is almost invariably produced. The child should be greased and wiped. By this means it is cleansed more thoroughly than by washing. Do not allow the flannel band to be put on too tight. If the bowels be costive a little sweet oil internally, and some rubbed over the abdomen, will be sufficient. If retention of urine, a hot flannel over the hypogastrium and a cold iron to the feet will produce enuresis. The napkins should be washed with soap, containing no irritating material, else excoriations may be produced.

Dr. Lynch: The time of getting up we cannot fix in all cases. The womb is large and heavy, and its supports are relaxed, and there is danger of flexion or lapse of the organ if the woman gets about too soon, but I do not think it necessary to keep her in bed twenty-five days. My rule is that as soon as the womb cannot be felt above the pubis getting up may be safely allowed. I think the milk fever is an entity, especially in the primipara. A woman may be doing well, and the next visit you find high fever, the breasts congested, hot and painful, and learn that she had a chill previous to your visit.

That a slight cause may produce great constitutional disturbance is proved by the temperature in tonsillitis, frequently being as high as 104° Fahr.

Dr. Leonard: I have used chloroform frequently in labor, and usually without trouble. In one case (the woman had mitral regurgitation) the pulse fell from 65 to 40, and 35, the countenance became pallid, and an unfavorable result seemed probable, but by withdrawing the chloroform for a short time, and sprinkling aqua ammonia on the bed clothes and around the patient, so that it could be inhaled with the chloroform, the anæsthetic was resumed without trouble. I do not allow the child to be washed, but grease it with vaseline and have it wiped. I use no bandage on the child, and the cord shrivels up in about half the time. To tie the cord I prefer a flat tape.

Dr. Cathell: Every one has a method of his own. To prevent hemorrhage from the cord, I tie with a flat tape, and immediately after the child is washed I tie again, in the same spot. Immediately after laying the child aside, I examine for the placenta, and remove it as soon as possible. I always examine it carefully, and incidentally call the attention of the nurse, or some one else in the room, to the fact that it has all been removed. I think this good practice, as it saves the physician from suspicion of having allowed part to remain. I frequently leave the application of the binder to the nurse. I agree with Dr. Murray, that it gives great comfort to the woman. In regard to the soiled clothes, I have them removed as soon as the woman indicates that their removal is more desirable than rest. To judge of the discharge I direct that small pieces of white rag shall be used to wipe the genitals, four or five times a day. Frequently we cannot rely on the nurse's report, and by using the rag we have a sure indicator.

Dr. Brinton: I think that chloroform retards the labor pains. Sore nipples I always treat by placing a glass shell over them, and in two or three days they are well. Believe in plenty of good food. Think ergot a dangerous remedy; can recall two or three children who lost their lives from its improper use. I do not use the binder. Remove the placenta immediately after the birth of the child. Have the child washed, because it is customary to do so.

Dr. Taylor: I never hesitate to give ergot when the os is sufficiently dilated; give it in combination with cimicifuga, ʒ ss of each, and ʒ ij immediately after the head has passed the perineum.

Dr. Morris: I have given chloroform very little in labor; use it in cases requiring instrumental assistance, but not in ordinary labor. I agree with Dr. Brinton, that it retards the labor pains.

Dr. Lynch: I have given chloroform to every woman that would consent to take it, and the number almost equals the number of labor cases that I have attended. I have never seen the slightest injury produced by it. When the head is high up, the labor may be a little retarded, but after the head descends there is no change in the pains, and the woman will bear down while snoring from chloroform, so it does not retard the latter part of the second stage. As to hemorrhage, I have never seen but one case in which there was danger from hemorrhage, and I do not think chloroform was the cause. I do not think we are justified in withholding this precious boon, espe-pecially after the strong endorsement of Simpson and others of great experience.

Dr. Murray: There is not much difference between the opinions of the members and my own. I think Dr. Lynch makes a mistake, and confuses condensation of the womb with its decrease in weight. It cannot lose much in weight in two days. The retrograde changes are not complete under forty days, and until this fatty metamorphosis occurs there is danger in allowing a woman to get up. As to the milk fever the Dr. puts me in a false position. I grant that inflammation of the breast will produce high fever. I think this condition is not necessary.

Dr. Lynch: Is this not essentially so in primipara?

Dr. Murray: I think not. The suggestion of Dr. Cathell, that some one should see the placenta, is a good one. I can see how an unfriendly nurse might make mischief by reporting that a piece of afterbirth is retained. I think the binder should be applied by the physician, and not trusted to any one else. In the small number of cases in which I have used chloroform, I think the results support the opinion of Dr. Brinton.—*Medical and Surgical Reporter.*

